# Exploring the Journey of Zinc Oxide Nanoparticles (ZnO-NPs) toward Biomedical Applications

**DOI:** 10.3390/ma15062160

**Published:** 2022-03-15

**Authors:** Fahadul Islam, Sheikh Shohag, Md. Jalal Uddin, Md. Rezaul Islam, Mohamed H. Nafady, Aklima Akter, Saikat Mitra, Arpita Roy, Talha Bin Emran, Simona Cavalu

**Affiliations:** 1 Department of Pharmacy, Faculty of Allied Health Sciences, Daffodil International University, Dhaka 1207, Bangladesh; fahadulislamdiu@gmail.com (F.I.); md.rezaulislam100ds@gmail.com (M.R.I.); aklima.ph@diu.edu.bd (A.A.); 2 Department of Biochemistry and Molecular Biology, Faculty of Life Science, Bangabandhu Sheikh Mujibur Rahman Science and Technology University, Gopalganj 8100, Bangladesh; sheikhshohag.bmb@gmail.com (S.S.); jalalrana86@gmail.com (M.J.U.); 3 Faculty of Applied Health Science Technology, Misr University for Science and Technology, Giza 12568, Egypt; mohamed.nafady@must.edu.eg; 4 Department of Pharmacy, Faculty of Pharmacy, University of Dhaka, Dhaka 1000, Bangladesh; saikatmitradu@gmail.com; 5 Department of Biotechnology, School of Engineering & Technology, Sharda University, Greater Noida 201310, India; arbt2014@gmail.com; 6 Department of Pharmacy, BGC Trust University Bangladesh, Chittagong 4381, Bangladesh; 7 Faculty of Medicine and Pharmacy, University of Oradea, 400087 Oradea, Romania

**Keywords:** ZnO-NPs, traditional synthesis, green synthesis, biomedical applications, toxicity

## Abstract

The field of nanotechnology is concerned with the creation and application of materials having a nanoscale spatial dimensioning. Having a considerable surface area to volume ratio, nanoparticles have particularly unique properties. Several chemical and physical strategies have been used to prepare zinc oxide nanoparticles (ZnO-NPs). Still, biological methods using green or natural routes in various underlying substances (e.g., plant extracts, enzymes, and microorganisms) can be more environmentally friendly and cost-effective than chemical and/or physical methods in the long run. ZnO-NPs are now being studied as antibacterial agents in nanoscale and microscale formulations. The purpose of this study is to analyze the prevalent traditional method of generating ZnO-NPs, as well as its harmful side effects, and how it might be addressed utilizing an eco-friendly green approach. The study’s primary focus is on the potential biomedical applications of green synthesized ZnO-NPs. Biocompatibility and biomedical qualities have been improved in green-synthesized ZnO-NPs over their traditionally produced counterparts, making them excellent antibacterial and cancer-fighting drugs. Additionally, these ZnO-NPs are beneficial when combined with the healing processes of wounds and biosensing components to trace small portions of biomarkers linked with various disorders. It has also been discovered that ZnO-NPs can distribute and sense drugs. Green-synthesized ZnO-NPs are compared to traditionally synthesized ones in this review, which shows that they have outstanding potential as a potent biological agent, as well as related hazardous properties.

## 1. Introduction

Nanotechnology is a rapidly developing discipline of science and technology concerned with producing and developing nanomaterials with particle sizes ranging from 1 to 100 nanometers [1]. Recently, the scientific research community worldwide expressed interest in synthesizing metal and metal oxide nanoparticles (NPs) [2]. The ZnO-NPs are of huge importance due to their wide variety of applications in photocatalysis, antimicrobial defense, and water purification. ZnO-NPs display properties that are distinct from those of typical NPs [3]. Additionally, these NPs are employed in the cosmetics industry to produced sunblock creams, which guard the human body against ultraviolet radiations [4]. Due to ZnO-NPs’ characteristics, such as their biocompatibility and non-toxicity, they are particularly well-suited for specialized biomedical applications [5,6,7]. Metal oxide NPs are important components in a wide range of consumer goods, including electronic equipment and cosmetics. ZnO-NPs are versatile materials with distinct chemical, optoelectronic, and wettability properties. They are easily made and widely used in a variety of industries, including wastewater treatment [8].

ZnO-NPs are manufactured using nanotechnology and are extensively used in various nanotechnology disciplines involving gas sensors [9], biosensors [10,11], cosmetics [12], ceramics [13], optical devices [14], display window materials for solar cells [15], and drug delivery [16,17]. Solar cells may directly transform light energy into electricity with their photovoltaic impact on ZnO-NPs [18].

ZnO-NPs absorb and scatter light very efficiently, making them excellent materials for optoelectronics applications that operate in the ultraviolet and visible spectrum areas. ZnO-NPs offer excellent photoluminescence properties, making them suitable for emission display systems, such as televisions [14]. In terms of photocatalytic degradation, ZnO-NPs seem to be the most promising choices [19]. The detection of gas leakage and the checking of gaseous contaminants in the environment may both benefit from semiconductor nano ZnO gas sensors [9]. ZnO-NPs are used to protect fabrics and wood from UV damage [20]. ZnO-NPs are made in a way that does not harm the environment, and they can control harmful microbes. Moreover, ZnO-NPs may be utilized as a treatment activator and a cross-linking agent in rubber treating, and can promote the vulcanization procedure in rubbers used to produce industrial and medical gloves, balloons, tires, and other rubber goods [21]. These substances have excellent antimicrobial and UV absorption properties and are commonly utilized in sunscreens, lotions, and ointments because of their versatility [12]. Antimicrobial ZnO-NPs are used in food and in can linings to keep fish, pork, peas, and maize safe from spoilage. ZnO-NPs have been proposed for next-generation biological applications, such as the delivery of medication, use as antimicrobial agents, and use as bioimaging probes [22].

**Figure 1:**
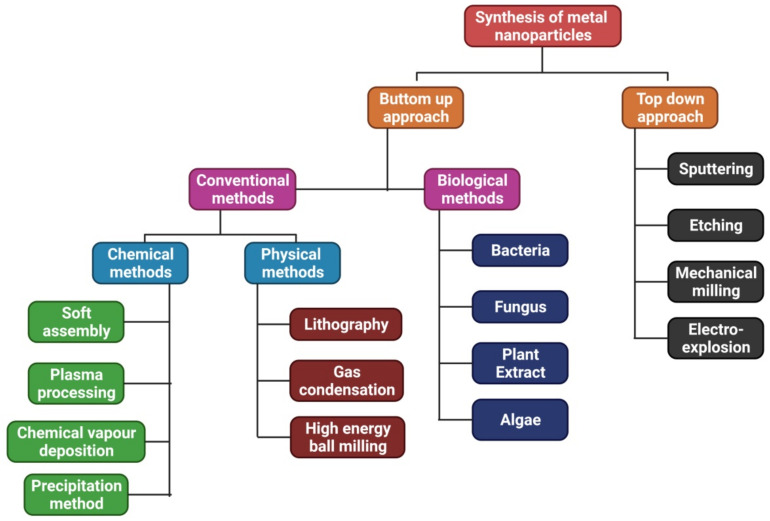
Methods to synthesize NPs from the bottom up and the top down.

The two ways that can be used to synthesize NPs are the top-down approach and the bottom-up approach (Figure 1). Electro-explosion, etching, sputtering, and mechanical milling are examples of top-down approaches, whereas bottom-up approaches comprise three basic methods for producing NPs: physical, chemical, and biological processes [23,24]. It is possible to produce pure, high-quality nanoparticles using conventional methods. Still, the process is expensive and sometimes results in the development of hazardous byproducts that may have detrimental consequences when employed for medical purposes. Furthermore, additional capping and stabilizing chemicals are required for these procedures [25]. This problem exists when NPs are produced using the green pathway, a bottom-up strategy that results in an oxidation/reduction reaction [26].

Green synthesis can be accomplished using plants, bacteria, fungi, and algae. They enable the significant manufacturing of pure ZnO-NPs [27]. During green synthesis, a mixture of different parts of medicinal plants is used to produce NPs. The phytochemicals play a role as a biocatalyst, capping agent, and organic stabilizer for NPs [28]. The process does not require high temperatures, pressures, expensive tools, or toxic chemicals [29]. The green synthesis of NPs is more cost-efficient, toxic-free, and environmentally beneficial than the expensive and hazardous procedures used before [30,31].

This review highlights the prevalent traditional method of generating ZnO-NPs, as well as its harmful side effects, and discusses how it might utilize an eco-friendly green approach. The study’s primary focus is on the potential biomedical applications of green-synthesized ZnO-NPs.

## 2. Methodology

**Figure 2:**
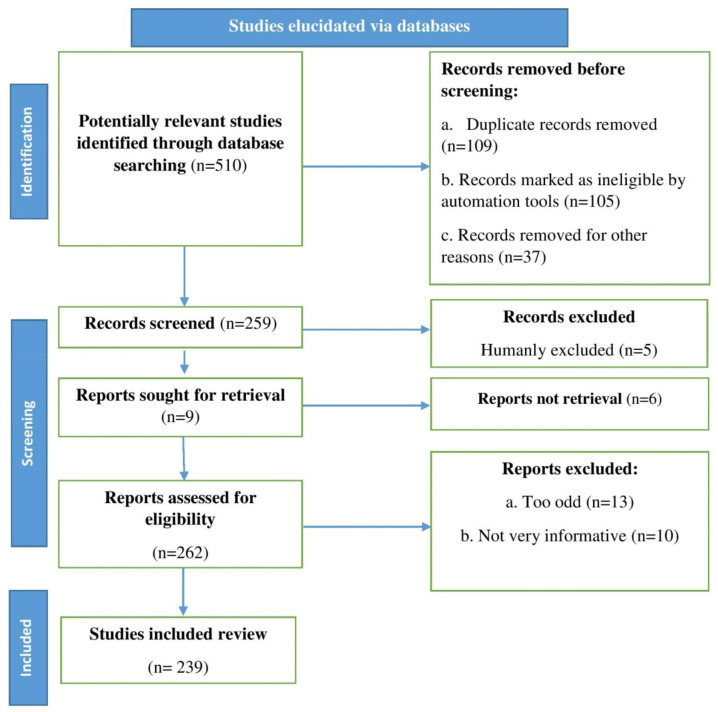
Stages involved in selecting published data for inclusion in the current study are depicted in a flow chart; n = number of literature reports.

To identify the most relevant articles (available in the most well-known medical/biology/chemical databases, such as Scopus, PubMed, and Web of Science) for this review as precisely as possible, “zinc oxide nanoparticles,” “traditional techniques,” and “biomedical applications” were used as primary keywords, and “plant extracts” and “green synthesis” were used as secondary keywords. An algorithm shown by the flow chart displayed in Figure 2 (according to the recommendations of Page et al. [32,33]) was used, which inserted all of the steps/selections requisite for identifying the necessary material in the literature.

## 3. Traditional Synthesis of ZnO Nanomaterials

Traditional methods for producing metallic NPs, such as ZnO-NPs, include mechanochemical and chemical processes. Sol-gel, hydrothermal, microemulsion procedures, and co-precipitation are all considered classic chemical synthesis approaches. Mechanochemical synthesis includes high-energy ball milling and laser ablation techniques [34,35,36,37,38]. The benefits and drawbacks of typical ZnO-NPs synthesis methods and particular innovative and noteworthy examples are briefly explored in the following sections of this paper.

### 3.1. Sol-Gel Technique

**Figure 3:**
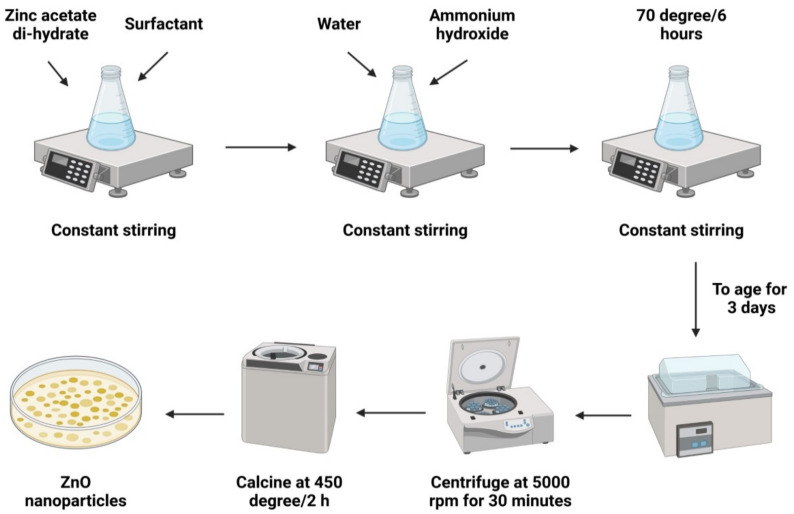
Diagrammatic representation of the stages required for the synthesis of metallic NPs (for example ZnO-NPs) employing the sol-gel process.

The transformation of a sol (e.g., a solution comprising inorganic metallic salts) progressively into a solid “gel” phase over a succession of hydrolysis and polymerization reactions is most commonly utilized to synthesize metal oxide NPs. Subsequently, the gel is treated to vaporize the solvents and heated to produce the final product [39,40,41]. Figure 3 depicts the sol-gel process in a simplified form. Using the sol-gel technique, it is possible to produce ZnO-NPs in a fine powder-like structure with a controlled chemical composition [42,43]. This process also has inherent drawbacks, including shrinkage, breaking while drying, and an inability to manage porosity [41]. Since the protocol is easy to follow and the critical material is generated quickly, it is frequently discussed in the relevant literature. Despite its shortcomings, this technique remains one of the most regularly employed. Zinc acetate dehydrates (Zn(CH_3_COO)_2_.2H_2_O), and ethanol were utilized as solvents to create rod-shaped ZnO-NPs in the range of 81.28–84.98 nanometers [23].

ZnO-NPs that averaged 28 nm in diameter with a spherical structure was produced by Jurablu et al. [44] using the sol-gel process. Zinc sulfate heptahydrate (ZnSO_4_.7H_2_O) and diethylene glycol (C_4_H_10_O_3_) surfactants were utilized in this method. Additionally, ZnO-NPs with a mean particle size between 12 and 30 nm were produced using a mixture of ammonia and methanol, as well as Zn(CH_3_COO)_2_.2H_2_O as the precursor in a sol-gel process, which resulted in spherical ZnO-NPs in the range of 50–60 nm [43,45].

### 3.2. Hydrothermal Technique

**Figure 4:**
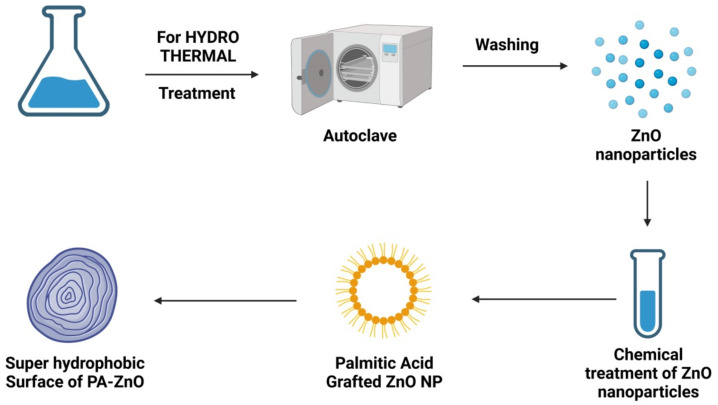
Diagrammatic representation of the stages required for metallic ZnO-NPs synthesis employing the hydrothermal technique.

An autoclave is a closed reaction vessel with high pressure and high temperature, where hydrothermal activities are carried out. Under high temperature and high pressure, the insoluble or challenging-to-dissolve materials are dissolved [46]. Various solvents can be utilized in these reactions, such as water or organic solvents such as ethanol or polyols, known as hydrothermal or solvothermal techniques, respectively [47,48]. An example of a hydrothermal procedure is shown in Figure 4. In addition to high product purity and crystallinity, hydrothermal methods control the final nanostructure size, shape, and crystal phase with little pollution due to the closed system environment [37,48]. Since the procedure is deemed environmentally beneficial, it is included in the green methods for producing ZnO-NPs. This method has some negatives; for example, it requires an extremely expensive autoclave and it has limitations for studies because the reactor cannot be kept open. There are also potential safety hazards throughout the autoclave procedure, which can be a concern [35,49,50]. Hydrothermal/solvothermal techniques, like the sol-gel strategy, are simple to start up. Examples include a study by Bharti and Bharati [51], which used a hydrothermal method to manufacture a length scale of 15.8–25 nm ZnO-NPs and various morphologies. ZnO-NPs with cylinder-shaped pores ranging from 9 to 12 nm in width were also manufactured by Reddy et al. [52], with the help of zinc nitrate hexahydrate (Zn(NO_3_)_2_.6H_2_O) and sodium hydroxide (NaOH). Similarly, by utilizing an autoclave unit, Wirunmongkol et al. [52] produced ZnO-NPs in which NaOH and Zn(NO_3_)_2_.6H_2_O were used as the initial precursors. Shaped like tiny prisms and flowers, the NPs were between 30 and 80 nm wide and 0.5–0.1 μm long, depending on the type of material used to make them.

### 3.3. Co-Precipitation Technique

**Figure 5:**
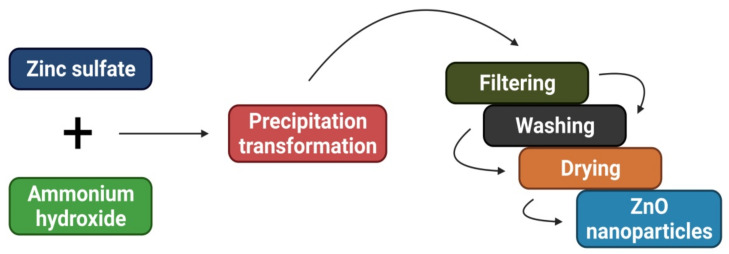
Diagrammatic representation of the stages required for ZnO-NPs synthesis employing the co-precipitation method.

The co-precipitation technique creates metallic NPs by simultaneous nucleation followed by growing and then agglomerating tiny nuclei. The co-precipitation process is depicted in Figure 5. This process has several advantages, including ease of use, minimal need for high temperatures, and ease of overall energy management [35]. As a side note, this approach has one significant drawback: it produces NPs with large quantities of water molecules attached to them [53]. Additionally, batch-to-batch repeatability issues, a wide range of particle sizes, and severe agglomeration are negatives [35,54,55]. However, remarkable instances include zinc acetate solution in methanol, in which spherical ZnO-NPs were synthesized by co-precipitation ranging between 2 and 10 nm in particle size. In contrast, a co-precipitation method using zinc acetate dihydrate, hydrochloric acid, and ammonia as reactants was used to manufacture ZnO-NPs. The ZnO-NPs was discovered to have a pseudo-spherical form with an average particle size between 11 and 20 nm [56,57]. A similar co-precipitation approach was used by Adam et al. [58] to produce ZnO-NPs with an average diameter of 140 nm.

### 3.4. Microemulsion Technique

**Figure 6:**
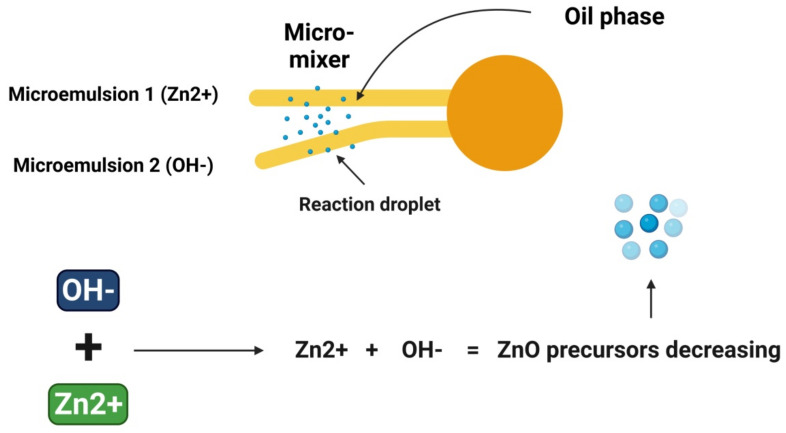
Diagrammatic representation of the stages required for metallic ZnO-NPs synthesis employing the microemulsion method.

Water droplets colliding with each other in a microemulsion environment resulted in a precipitation reaction, which led to the formation of NPs with surfactant-stabilized nucleation. The microemulsion process is depicted in Figure 6. The rewards of this approach include its simplicity, thermodynamic stability, and low accumulation. Microemulsion techniques have several drawbacks, including the impact of temperature and pH on the stability of the microemulsion and the persistent demand for highly concentrated surfactants and/or cosurfactants that may irritate [35]. ZnO-NPs have been manufactured in microchannel reactor systems with an average diameter of 16 nanometers by Wang et al. [59]. Following a drying period of 2 h at 130 °C, the ZnO-NPs were then calcined at 550 °C for 3 h. ZnO-NPs were also produced by Li et al. [60] via a simple procedure of microemulsion, with diverse morphologies including columnar and spherical.

### 3.5. Laser Ablation Technique

**Figure 7:**
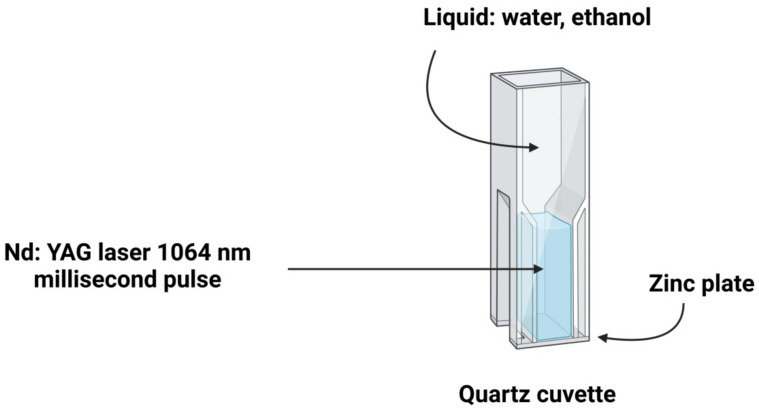
Diagrammatic representation of the stages required for metallic NPs synthesis (for example ZnO-NPs) employing laser ablation.

A typical laser ablation technique can remove metallic ions from metal surfaces by employing a laser beam and a small liquid quantity of methanol, ethanol, and purified water. The surface is immersed in the liquid. A schematic representation of the laser ablation approach is shown in Figure 7. Simplicity, and a quite safe procedure from an environmental standpoint, are two of the approach’s advantages, resulting in a process that is both efficient and simple to carry out [61]. Pyrolysis byproducts (the result of laser ablation when organic substances are present) have yet to be fully clarified and need to be addressed [62]. The works of Al-Dahash et al. [63] are exciting: with laser ablation in NaOH aqueous solution, they could produce ZnO-NPs from 80.76 to 102.54 nm with a spherical structure. In addition, Farahani et al. [64] used a zinc target in a solution of methanol and distilled water to generate ZnO-NPs with a roughly spherical morphology ranging from 1 to 30 nm by laser ablation. In the same way, Mintcheva et al. [61] indicated that they made ZnO-NPs that were rod-shaped, 30 nm in diameter, and 40–110 nm in size.

### 3.6. High-Energy Ball Milling Techniques

**Figure 8:**
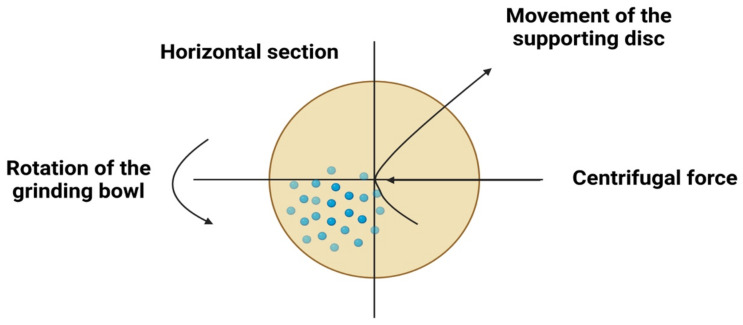
Diagrammatic representation of the stages required for metallic NPs synthesis (for example ZnO-NPs) employing high-energy ball milling procedures.

The high-energy ball milling technique is a manufacturing process that produces fine metal NPs in an elevated shaker mill [65]. This technology is depicted in Figure 8. Its key advantage is the ability to generate vast quantities of material simultaneously. Its downsides include contaminants from milling balls and/or from the environment and irregularly shaped NPs that result from this process [66,67,68]. ZnO-NPs may still be synthesized using commercially available ZnO powder with a mean of 0.8 m particle size, as demonstrated by Prommalikit et al. [69], who used high-energy ball milling to manufacture ZnO-NPs. Particles with a final size of 200–400 nm were obtained through milling. In a similar vein, Mohammadi et al. [70] synthesized rod-shaped ZnO-NPs in the 20–90 nm range using a high-energy ball milling technique. Salah et al. [70] employed the same high-intensity ball milling procedure to make ZnO-NPs from ZnO microcrystalline powder. The samples were ground into a ball mill for 2, 10, 20, and 50 h. The size of the particles changed over time, according to the results. The smaller the particle size is, the longer the ball milling process lasts. Spherical ZnO-NPs with approximately 30 nm particle sizes were found in the milled sample.

## 4. Green Synthesis of ZnO-NPs

### 4.1. Green Synthesis of ZnO-NPs Using Plant Extract

Because of the unique phytochemicals that they generate, plant components, for instance, the root, stem, leaf, seed, and fruit, have been employed to fabricate ZnO-NPs. The use of organic isolates of plant parts is a highly eco-friendly, cost-efficient method that does not need intermediary base groups. It takes a fraction of the time, requires no expensive equipment or precursors, and produces a highly natural and magnitude-enriched product devoid of contaminants [71]. Plants are considered a popular source of NP synthesis because they allow for the significant production of NPs with various shapes and sizes [72].

Phytochemicals, such as polysaccharides, vitamins, alkaloids, polyphenolic compounds, amino acids, and terpenoids released by plants, decrease metal oxides or metal ions to around 0 valence metal NPs [71,72]. The plant portion’s manufacturing of ZnO-NPs extracted from flowers or leaves is mainly processed via being bathed in running tap water and sterilized double distilled water. The plant portion is then allowed to dry at room temperature before being weighed and crushed with a mortar and pestle. The necessary amount of Milli-Q H_2_O is added to the plant component and boiled under vigorous agitation using a magnetic stirrer [71,72,73,74,75]. The plant’s extractions are made by filtration through Whatman filter paper (sample). To ensure efficient mixing, the mixture is heated to the necessary temperature for the necessary time to integrate the extract into 0.5 mm of hydrous zinc sulfate or zinc nitrate, or ZnO or solution [74,75]. At this point, some experiments were done with extract concentration, temperature, duration, and pH to see what works best. An incubation period causes the mixture to turn yellow as visual proof of the newly produced NPs [74,75].

Next, the mixture is centrifuged and dried in a hot oven to obtain the crystal NPS from the synthesized NPs, and confirmed by UV-Vis spectrometry [76]. To further characterize the synthesized NPs, various techniques, such as Field Emission Scanning Electron Microscopy (FE-SEM, JEOL IT800 series, New York, NY, USA), X-ray Photoelectron Microscopy (XPS, Phadke Instruments Private Limited, Maharashtra, India), Energy Dispersion Analysis of X-ray (EDAX, Nunes Instruments, Tamil Nadu, India),Scanning Electron Microscopy (SEM, Analytical Technologies Limited, Gujarat, India), X-ray diffractometer (XRD, Expert Vision Labs Pvt. Ltd., Maharashtra, India), UV-Visible Diffuse Reflectance Spectroscopy (UV-DRS, Nunes Instruments, Tamil Nadu, India), Fourier Transform Infrared Spectroscopy (FTIR, Alliance Enterprise, Mumbai, India), Transmission Electron Microscopy (TEM, Expert Vision Labs Pvt. Ltd., Maharashtra, India), and Atomic Force Microscopy (AFM, V Instek Analytical, Gujarat, India), are propagated [75,76,77]. Microwave irradiation (MI, V Instek Analytical, Gujarat, India) takes less time than conventional heating (CH), according to an experiment by Jafarirad et al. [78], and this is due to the higher level of heating provided by MI and a consequently faster response rate. *Anisochilus carnosus* [79], *Plectranthus amboinicus* [80], and *Vitex negundo* [81], members of the Lamiaceae family, have been widely investigated; the size of produced NPs reduces as the content of a plant extract increases [79,80,81].

Additionally, results comparing the size ranges recorded using other techniques, such as FE-SEM, TEM, and XRD, revealed similar range values [80,81]. SEM and EDAX yielded results that differed slightly from those of XRD. According to the Debye-Scherrer equation, NPs synthesized from the leaves and flowers of *Vitex negundo* had the same diameter of 38.17 nm, validated by XRD analysis [81]. For the fabrication of ZnO-NPs, the leaves of the *Azadirachta indica* of the Meliaceae family were the ones most typically employed [82,83]. XRD and TEM examination verified that the NPs in all trials were in the same size range, with spherical and hexagonal disc-shaped NPs and Nano buds carboxylic acid, alkane, amine alcohol, carbonate moieties, and amide were involved in the synthesis of NPs, as evidenced by FTIR investigations. *Aloe vera* leaf extract and leaf peel belong to the Liliaceae family [84,85]. The size of synthesized NPs differed (NPs synthesized from peel were more extensive, as validated by SEM and TEM studies), but the forms were similar (hexagonal and spherical). *Agathosma betulina*, *Pongamia pinnata*, *Plectranthus amboinicus*, *Nephelium lappaceum*, and *Calatropis gigantea* were extracted for synthesized NPs, which form aggregates [86]. Plants employed to synthesize ZnO-NPs up to these points are included in Table 1.

### 4.2. ZnO-NPs Green Synthesis Using Bacteria

There are various drawbacks to employing bacteria to synthesize NPs, including the time and effort necessary to screen microbes, the need for constant observing of culture broth and the entire process, the NPs’ shape and size, and the expense of the media used to grow bacteria. Using an eco-friendly technique, the photocatalytic activity and degradation of nanoflowers ZnO were demonstrated by *B. licheniformis*. The photocatalytic action for these nanoflowers was shown to be improved when compared to existing photocatalytic materials. It has been speculated that the more considerable oxygen vacancy in the produced NPs provides this property. It is possible to employ photocatalysis as a bioremediation method because it generates active species by absorbing light. Synthesized nanoflowers based on *B. licheniformis* were 40 nm wide by 400 nm long [106].

*Rhodococcus* can persist in unfavorable conditions and metabolize hydrophobic substances, which enables it to contribute to biodegradation [107]. *Rhodococcus pyridinivorans* and zinc sulfate were used to manufacture spherical NPs with a 100–130 nm size range, which XRD and FE-SEM assessment confirmed. In addition, FTIR examination indicated the existence of mononuclear benzene band, secondary sulfornamide, lactone, amine salt, monosubstituted alkyne, enol of 1-3-di ketone, hydroxy aryl ketone, amide I bending band, alkane, amide II stretching band, and phosphorus compound [108]. NPs of ZnO were created using *Aeromonas hydrophilla* as a substrate for ZnO synthesis. AFM and XRD analyses showed that the NPs produced had a size range of 42–64 nm and diverse forms including oval and spherical [109]. Because it is difficult for rhamnolipid to make micelle aggregate on carboxymethyl cellulose, this helps keep ZnO-NPs from breaking apart into micelle groups, making them more stable [110]. Because of its lengthy carbon chain, it works as a better capping agent [111]. The TEM, XRD, and DLS analyses revealed the synthesis of spherical NPs with a nano size range of 27–81 nm [111]. The properties of ZnO-NPs produced utilizing bacterial strains are shown in Table 2.

### 4.3. ZnO-NPs Green Synthesis Using Microalgae and Macroalgae

Unicellular algae (chlorella) and multicellular algae (chlorophyll) are examples of photosynthetic organisms (for instance, brown algae). Basic plant structures, such as leaves and roots, are absent from algae. Marine algae are classified according to the pigments they contain, such as Rhodophyta, Phaeophyta, and chlorophytes, which have red, brown, and green pigments, respectively. For the formation of Au and Ag NPs, algae have been extensively exploited. However, their utilization for ZnO-NPs synthesis has been limited and documented in relatively few works [92]. The potential of microalgae to break down hazardous metals and transform them into less harmful forms has drawn significant attention [116]. *S. muticum* and *S. myriocystum*, both Sargassaceae plants, were employed to synthesize ZnO-NPs. Sulfated polysaccharides were present in the NPs investigated by XRD and FE SEM, revealing similar NP sizes and hexagonal wurtzite structure. For *S. myriocystum*, DLS and AFM measurements demonstrated varied size ranges, with carbonyl and hydroxol stretching in NPs that vary substantially in form [99]. The micro- and macro-algae listed in Table 3 were used to synthesize ZnO-NPs.

### 4.4. ZnO-NPs Green Synthesis Using Fungus Theorem

Extensive production, easy downstream processing, and commercial feasibility make extracellular NPs from fungi beneficial [120]. Because of their higher tolerance and their ability to bioaccumulate metals, fungi are preferred over bacteria [121]. Mycelia of *Aspergillus fumigatus* were used to produce ZnO-NPs. According to the DLS study, NPs ranged from 1.2 to 6.8 in area size, with a 3.8 average size. AFM established the average height of NPs to be 8.56 nm for 90 days, with a significant particle size of more than 100 nm. After 90 days, they developed an agglomeration with an average particle size of 100 nm, indicating that the produced NPs were stable for 90 days [122]. SEM confirmed a size range between 54.8–82.6 nm for NPs produced from *Aspergillus terreus* that belong to the Trichocomaceae family. XRD investigation results revealed a 29 nm average size, which was determined using the Debye-Sherrer equation. FTIR analyses indicated the formation of primary alcohol, aromatic nitro compounds, and amine in the produced NPs [123]. SEM, TEM, and XRD analysis verified that NPs generated with *Candida albicans* had a comparable size range of 15–25 nm [124]. In most cases, ZnO-NPs developed from *Aspergillus* species were spherical. Table 4 lists the fungi most typically employed for ZnO-NPs production

### 4.5. ZnO-NPs Green Synthesis Using Other Green Sources

NPs can be synthesized using biocompatible chemicals and alternative green sources. NP nucleation and synthesis reactions can be carried out within a short time and cost-efficiently. They result in the production of NPs with a well-dispersed nature that may be precisely regulated in shape and size [127]. Antibacterial capabilities improved in 99.9% of NPs produced using a wet chemical method when layered on cotton fabric [128]. Table 5 summarizes a few more green resources used to synthesize ZnO-NPs.

## 5. Biomedical Applications of Green-Synthesized ZnO-NPs

**Figure 9:**
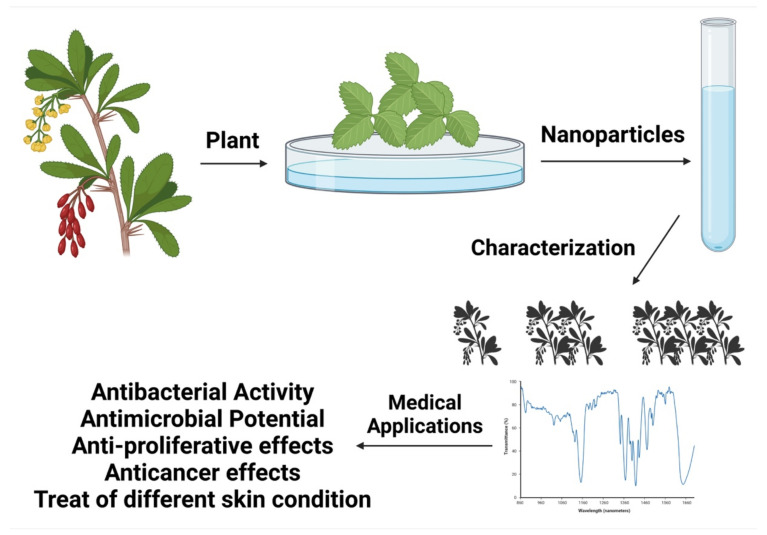
An illustration of the green synthesis and use of ZnO-NPs.

There has been a sharp rise in attention to NP research in the past decade, particularly in regard to biological applications [132]. Since nanotechnology has been integrated into medical research, a more excellent grasp of molecular biology has been achieved. As a result, innovative treatment strategies may be possible for illnesses that were previously impossible to address due to size limits [133]. For biomedical applications, the formulation of biofunctional NPs has attracted various research groups that are continually addressing this subject [134]. Biomedical applications of ZnO-NPs are now under investigation using a wide range of materials and chemical synthesis processes, as we have discussed in this study. As an ecological element and part of nature’s intrinsic materials, zinc has a vital role in human, animal, and plant metabolism. Zinc is required for all living species, which must be exposed to environmentally appropriate amounts of zinc in the biosphere. ZnO is extensively utilized in cosmetic, pharmaceutical, and medicinal applications, and as a nutritional supplement. Even though ZnO dust and fumes are typically considered harmless, breathing them should be avoided. Regulations have been put in place to limit the risk of exposure [135]. Figure 9 depicts the green production and uses of ZnO-NPs.

### 5.1. ZnO-NPs Antibacterial Activity

Organic and inorganic materials are the most common divisions in pharmaceutical medicinal agents. Organic medicinal drug substances have been found to be less stable at high temperatures and high pressures, when compared with inorganic medicinal drug substances [136]. ZnO-NPs are powerful pharmacological agents for therapeutic applications. ZnO-NPs seem to have a significant therapeutic drug activity when compared with microparticles. It is noteworthy that the specific mechanisms of medicinal drug action have not been wholly established [137]. Both gram-positive and gram-negative bacteria are germicidal to ZnO-NPs [138], and the ZnO-NPs also include medical therapeutic actions against high temperature and high pressure-resistant spores. Research shows that their extent and concentration influence ZnO-NPs’ medicinal properties, but not their crystalline structure or particle type. Therefore, the more NPs there are, the more potent the medical medicine [139].

Synthesized ZnO-NPs, which have natural antibacterial effects and are photocatalytic in the ultraviolet (UV-B) light range, can create potent hydroxyl (-OH) free radicals to kill dangerous pathogens and germs at wound sites [140]. This observation led to the development of a 3D printed customized wound-healing template made of ZnO-NPs that were uniformly scattered within an alginate template, which can be easily created and contour-printed to the exact size and depth of a wound. 3D printing consist of the adding of material layer by layer, allowing for the fabrication of unique shapes and customizability, which are crucial in biomedical areas such as tissue engineering and pharmaceuticals [141].

ZnO-NPs’ medical medication action mechanism is still a mystery. Hydrogen peroxide emission may be the essential factor in the action of therapeutic drugs. It is also possible that the mechanism is due to the binding of particles on the bacterial surface, owing to static tensions [142]. According to the results, the antibacterial activity of ZnO-NPs seems to be stronger than that of tiny particles. Particle dosage, treatment duration, and the NP production process influence NPs’ efficacy. Furthermore, the surface area and the size of particle variation, which are noteworthy in green-synthesized ZnO-NPs, are responsible for enhanced antibacterial activity. Future medical difficulties might benefit from green-synthesized ZnO-NPs applications in food safety and agriculture that have not yet been confirmed [143]. Table 6 provides applications of green-synthesized ZnO-NPs for antibacterial purposes.

### 5.2. ZnO-NPs Antimicrobial Potential

ZnO is explored as a potential drug carrier in micro-and nanoscale formulations. Even though the medicament-specific mechanisms are not fully understood, it has been proposed that the ROS produced on the particle’s surface, membrane dysfunction, zinc ion release, and the NPs’ acquisition area unit are the common causes of cell swelling. Management of ZnO-NPs at elevated temperatures significantly affects their therapeutic activity, whereas treatment at lower temperatures reduces activity. The mechanisms underlying ZnO-NPs’ medicament activity are unknown. While it is hypothesized that oxide generation contributes to such activity, it is indicated that the binding between particles and microorganism surface, due to electrical forces, could be a mechanism for ZnO-NPs’ medicament behavior. This could be accomplished using oxygen electrode analysis and chemiluminescence. Metal NPs are highly ionic and can be generated with exceptional crystal and high surface, and morphologies with varying edge/corner and reactive surface sites. The ZnO-NPs area unit is subject to current research concerning therapeutic procedures with ablation regimens. Despite having a more significant thermal effect on neoplasm ablation, NPs will provide an antineoplastic medical specialty with a synergetic anticancer impact at the time of heat presence. They may even be imaged to achieve precise medical assistance. Numerous experiments revealed that understanding the molecular mechanism underlying tumor-mediated NP ablation will aid in the development of NPs with appropriate composition and characteristics to induce the ablation property [159,160,161].

### 5.3. Proliferating Cells Selective Killers

ZnO cancerous cells are killed by ZnO-NPs, whereas healthy cells are unaffected [162,163]. Before ZnO-NPs can be used medically, a slew of issues must be addressed, including a lack of biocompatible dispersion procedures and a more profound knowledge of the mechanism underlying their selective cytotoxicity [142]. To date, there have only been a few investigations on the ZnO-NPs cytotoxicity mammal cells, and experts are divided about the importance of the results that have been published. A study found that ZnO-NPs have no influence on T cells’ viability in both gram-negative and gram-positive microorganism concentrations [164]. According to various publications, these NPs are harmless with respect to the culture of human dermal fibroblasts; still, they are harmful to metastatic tumor cells [165] and the cells of vascular endothelial [166], triggering programmed cell death in neural stem cells. It has been stated that the NPs’ size can affect cell viability. Jones et al. [34] discovered that ZnO-NPs with a diameter of eight nm were more hazardous than were larger zinc oxide particles (50–70 nm) in *Staphylococcus aureus*. Hanley et al. [167] recently established a reverse relationship between class cells’ toxicity and NP size, such as reactive oxygen species (ROS) production. In contrast, Deng et al. [168] showed ZnO-NPs’ toxic influence on nervous stem cells in a dose-dependent manner, regardless of particle size.

### 5.4. ZnO-NPs Anticancer Effects

ZnO cancer nanotechnology has vast implications for molecular identification, molecular imaging, and tailored medical treatment, according to the nursing knowledge domain area of analysis in engineering, science, and medications. To put it simply, nanometer-sized particles, such as semiconductor quantum dots and iron chemical complex nanocrystals, exhibit optical, magnetic, or structural features that are not found in molecules or bulk materials. As soon as these NPs are attached to antigen-targeting ligands, such as antibodies or peptides, they can target neoplasm antigens as biomarkers as well as neoplasm vessels with significant similarity and specificity. Because of their large surface areas and functional groups, many diagnostic and therapeutic substances can be conjugated to NPs in the 5–100 nm diameter range. A junction rectifier to bio-affinity NPs for molecular and cell imaging can provide customized medical treatment using NP medication. Researchers have recently developed and incorporated nano-devices to detect and screen cancer in early stages. Biomarkers for cancer diagnosis and treatment based on individualized molecular profiles and tailored genetic and super molecular biomarkers are now possible because of these breakthroughs in personalized medicine [169].

**Figure 10:**
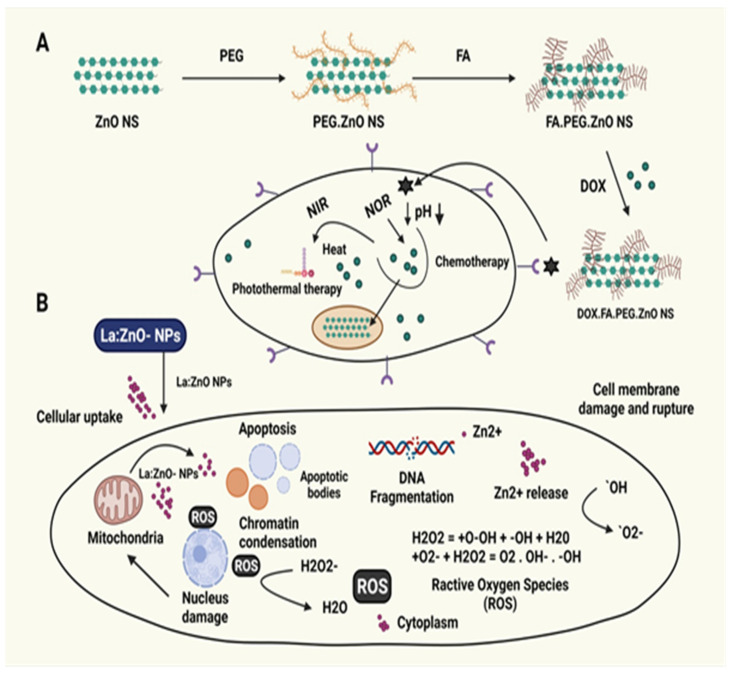
The mechanisms underlying the anticancer activity of green ZnO-NPs. (**A**) Cytotoxic action of La-doped ZnO-NPs causes cell death [195]. (**B**) The development of DOX-FA-ZnO NS is a unique breast cancer treatment drug delivery system [195]. ZnO nanostructures (ZnO-NS), doxorubicin (DOX), folic acid (FA), near-infrared (NIR), polyethylene glycol (PEG), and lanthanum (La) are all components of ZnO-NS.

Several types of research have indicated that ZnO-NPs positively influence cancer cell growth. It was found that the cell response to ZnO-NPs was dynamic. Hence, the final composition was affected by multiple challenging or intersecting signals in the microenvironment, as revealed by Premanathan et al. [142]. ZnO-NPs were more hazardous to HL60 cancer cells than to normal PBMCs with a therapeutic index, according to the findings (i.e., hepatotoxic dose) [142]. The inability to distinguish between traditional and changed tissues in malignant neoplasm medicine may be of essential clinical interest and the biggest hurdle in treatment [170]. Even though various commonly prescribed drugs can slow down the rate at which cells divide, many of these treatments have a low therapeutic index [171,172]. Table 7 summarizes the anticancer uses of ZnO-NPs synthesized by the green synthesis technique, whereas Figure 10 illustrates the molecular mechanisms underpinning green ZnO-NPs’ anticancer action.

**Table 7:** Anticancer applications of ZnO-NPs generated in the green synthesis process.

Platform	Raw Material	Size	System	Targeted Cell Line	Reference
Fungi-mediated	*Pichia kudriavzevii* yeast	10–61 nm	ZnO-NPs	MCF-7, breast	[173]
	*Penicillium chrysogenum* fungus	29–37 nm	ZnO-NPs	MCF-7, breast HCT-116, colon	[174]
	*Aspergillus niger* fungus	80–130 nm	ZnO-NPs	HepG2, liver	[175]
	*Aspergillus niger* fungus	11.8–17.6 nm	ZnO-NPs	A549, lung	[176]
	*Aspergillus terreus* fungus	28–63 nm	L-asparginase—ZnO-NPs	MCF-7, breast	[177]
Algae and plant-mediated	*Sargassum muticum* algae extract	30–57 nm	ZnO-NPs	HepG2, liver	[178]
	*Sargassum muticum* algae extract	50–100 nm	ZnO-NPs	WEHI-3, murine leukemia	[179]
	*Sargassum muticum* algae extract	3–8 nm	ZnO-NPs	PANC-1, pancreas CaOV-3, ovarian COLO205, colon HL-60, leukemia	[180]
	*Gracilaria edulis* algae extract	4.04 ± 1.81 nm; length 1.39 ± 0.6 nm; width	ZnO-NPs	SiHa, cervical	[181]
	*Rehmanniae radix* plant extract	10–12 m	ZnONPs	MG-63 bone	[182]
	*Myristica fragans* plant extract	100–200 nm	ZnONPs	HeoG2, liver	[183]
	*Albizia lebbeck* stem bark	66.25 nm	ZnONPs	MCF-7, breast MDAMB231, breast	[184]
	*Mangifera infica* leaves	45–60 nm	ZnO-NPs	A549, lung	[185]
	*Pongamia pinnata* seeds	30.4–40.8 nm	ZnO-NPs	MCF-7, breast	[186]
	*Eclipta prostrata* leaves	20–1.3 nm	ZnO-NPs	HepG2, liver	[187]
	*Borassus flabellifer* fruit extract	110 nm	ZnO-NPs loaded with DOX	MDAMB231, breast	[188]
	*Ziziphus nummalaria* leaves	17.33 m	ZnO-NPs	HeLa, cervical	[189]
	*Laurus nobilis* leaves	47.27 nm	ZnO-NPs	A549, lung	[152]
	*Nephelium lappaceum* peel	-	ZnO-NPs	HepG2, liver	[190]
	*Tecoma castanifolia* flower	70–75 nm	ZnO-NPs	A549, lung	[191]
	*Gymnema sylvestre,* plant extract	38 nm 33/27/23 nm	ZnO-NPs La/Nd/Ce—ZnO-NPs	A498, kidney	[158]
	*Costus pictus,* leaves	20–80 nm	ZnO-NPs	DLA, Daltons lymphoma ascites	[5]
Protein mediated	Collagen protein	20–50 nm	ZnO-NPs	HepG2, liver	[192]
	Milk casein protein	9.3–13.7 nm	ZnO-NPs loaded with curcumin	MCF-7, breast HeLa, cervical MDAMB231, breast MG-63, bone	[193]
	Tocopherol lipid	100 nm	Chitosan coated ZnO-NPs	HeLa, cervical	[194]

### 5.5. Treatment of Different Skin Conditions

ZnO is frequently used to treat skin diseases, including diaper rashes, and in shampoos, anti-dandruff treatments, hemimorphite creams, and antibacterial ointments. Additionally, it is a component of tape that athletes use as a bandage to prevent soft-tissue injuries during workouts [196]. It is possible to use ZnO-NPs in the form of an ointment, cream, or lotion to guard against UV-induced skin damage and the resulting sunburn. Only this UVA/UVB reflector, which is entirely photo-stable, has been authorized for use as a sunscreen [197]. As a sunscreen component, ZnO inhibits all UV-A (320–400 nm) and UV-B wavelengths’ ultraviolet radiation. Additionally, ZnO-NPs are considered to be common diverse conventional physical sun blocks, protecting pigments and area units that need to be free from irritations, allergens, and acne-causing properties [198].

### 5.6. Drug Delivery

Among several nanotechnology implementations, drug delivery via ZnO-NPs has developed into a highly effective method for treating various disorders such as cancer [199,200]. Nanomaterials are one of the essential mechanisms in the delivery of drugs. ZnO-NPs have been used for drug delivery for multiple diseases [199,200]. ZnO quantum dots were employed in a study by Yuan et al. to administer doxorubicin to HeLa cells [201]. ZnO-NPs were stabilized by encasing them in chitosan. According to the results of their study, this drug delivery method could be utilized to target cancer cells with doxorubicin [201]. It is also important to note that one of the primary uses of NPs is the transport of genetic material to distinct cells, particularly tumor cells [200]. This technology for gene delivery has several benefits. For instance, the appearance of a plasmid-encoded gene on NPs’ surfaces could assure reliable and effective gene delivery to the receiving tissues [199,200].

Consequently, NPs can be an effective instrument for directing genes to various cells, including tumor cells. Nie et al. [202] reported that they had created ZnO tetrapod-like nanostructures that might be employed as innovative gene-delivery vectors. They revealed that ZnO-NSs, such as a silica-coated amino-modified tetra pod, could bind effectively to DNA through electrostatic interactions, potentially increasing the efficacy of melanoma cell transfection [202]. In another investigation, Zhang et al. [203] showed that polycation-capped ZnO quantum dots might transmit DNA into COS-7 cells. Additionally, the usage of this method allows for the instantaneous visualization of gene delivery [203]. Several investigations have employed metal oxide NPs for gene silencing and gene delivery. However, it is vital that further knowledge be obtained [199,200]. ZnO-NPs-based drug delivery methods are shown in Table 8.

### 5.7. Bioimaging

ZnO is a common semiconductor material that can completely replace the typical Cd-related species found in biological and optical environments [199,217]. At this point, a variety of ZnO-NPs types have been identified. The bioimaging potential of ZnO-NPs is intriguing to researchers [199,217]. A wide range of biological and medicinal uses are possible for this feature. For instance, luminous ZnO-NPs may have excellent photophysical qualities [199,217]. The surfaces of these NPs have been demonstrated to be easily manipulated. For ZnO-NPs, it has been discovered that their quantum yield (QY) may be increased to about 30% following careful tweaking [199,200,217]. According to the common consensus, ZnO is a safe material. ZnO has been used in sunblock goods and in diet packing as a food preservative. This means that many biological and medicinal applications could use the luminous features of ZnO-NPs [199,200,217]. The bioimaging uses of ZnO-NPs are shown in Table 9.

## 6. Toxicity Associated with ZnO-NPs

ZnO is a nanomaterial that is widely employed in a variety of applications [227]. Using a well-known photocatalyst, the degradation of environmental pollutants has garnered considerable attention from researchers [19]. Zinc salts have been utilized as an active ingredient in lubricants for a long time [228] and used by the pharmaceutical industry to make emollients [229]. In wound care, anti-infection therapeutic goods, and disinfectants, ZnO-NPs containing medicines are extensively employed. ZnO-NPs have many applications in cosmetics, hair and skincare formulations, protective sunblocks, food additives, and vitamins, among others [230,231]. ZnO is used as an antibacterial compound commonly used in lotions, ointments, body washes, and surface coatings to prevent the growth of microorganisms [146,232]. As nutritional supplements, ZnO-NPs have also been utilized by humans and livestock to stimulate the body’s reaction to inflammation and to enhance the immune system [233]. The expanding use of ZnO-NPs in consumer goods and pharmaceuticals has prompted researchers to look into the potentially hazardous consequences of ZnO-NPs for human health [230]. The advantages must be carefully balanced against the potential disadvantages of other NPs.

According to the available research, the inhalation of ZnO-NPs has the most harmful effects on human lungs [230,234]. The size and surface area of ZnO-NPs have been linked to the severity of inflammatory illness caused by their exposure [235]. Previous research has shown that ZnO-NPs elicit a more severe inflammatory response than liquid zinc ions [236]. In various investigations, ZnO-NPs’ cytotoxic characteristics have been tested on human red and white blood cells. A cytotoxic effect has been seen at concentrations more than 50 ppm, likely due to increased oxidative stress [237,238]. At more significant concentrations than predicted in the environment, ZnO-NPs can produce acute impacts on fish [239]. Therefore, a thorough evaluation of ZnO-NPs’ characteristics, routes of administration, target cells, and related physiological processes is required to better understand the therapeutic advantages and to minimize unwanted harmful consequences and negative clinical diagnostic potential. Long-term effects must still be investigated for the better and safer use of these NPs.

## 7. Conclusions and Future Perspectives

Because of its environmentally friendly nature, the green synthesis of ZnO-NPs is favored. The use of diverse plant components, bacteria, fungi, and algae to synthesize ZnO-NPs is an efficient, simple, and environmentally friendly approach. Plant extracts contain a variety of biomolecules that act as reducing, capping, and stabilizing agents, including amino acids, proteins, and a variety of additional primary and secondary metabolites that serve as reducing, capping, and stabilizing agents during the synthesis process.

The synthesis of these critical nanomaterials has some risks for the environment and for civilization. As a result, the biological qualities of these materials are directly affected. The use of biomolecules and living organisms as nanomaterials’ capping agents in green nanotechnology is a powerful option as a potential solution to minimize the development of toxic products and undesirable reactions with various biologic membranes. NPs biogenesis with minimal impact on the environment has been the focus of research for the past decade. These NPs can be precisely sized and shaped using green synthesis methods. Medical practitioners are increasingly using antimicrobial NPs bandages. Medicine delivery and clinical diagnostics have produced a growing demand for these technologies. A rising number of people are interested in environmentally friendly nanomaterials such as ZnO-NPs, which can be produced with minimal danger and expense. Green synthesis technologies appear to be increasing in popularity in recent years. ZnO-NPs generated from plants may be an essential research topic in the biomedical sectors. The green synthesis of ZnO-NPs using plants and microbes has been highlighted in this review, as it is a rapid, simple, environmentally friendly, and relatively low-cost process. Biosynthesized ZnO-NPs for biomedical applications, especially against pathogenic germs, have also been addressed, to overcome the limitations of conventional chemical and physical methods. The biological source affects the size of ZnO-NPs and, consequently, their biological activities. However, additional study is needed to standardize synthesis procedures, as a critical limitation of green chemistry is the variability of the end products. Further in vitro and in vivo experiments are expected to elucidate the mechanism of action involved at the cellular level, with applications in various biomedical fields.

## Figures and Tables

**Table 1:** ZnO-NPs synthesized using a plant-mediated process.

Common Name	Plant (Family)	Extraction Part	Functional Group	Shape	Size (nm)	References
Coptis Rhizome	*Coptidis rhizoma* (Ranunculaceae)	Dried Rhizome	Primary and secondary amine, aromatic, aliphatic amine, alcohol, carboxylic acid, alkyl halide, and alkynes.	Spherical, rod-shaped	2.9–25.2 (TEM)	[87]
Neem	*Azadirachta indica* (Meliaceae)	Fresh leaves	Amine, alcohol, ketone, carboxylic acid	Spherical	18 (XRD)	[88]
Indian beech	*Pongamia pinnata* (Legumes)	Fresh leaves	O-H stretching, C=O spreading carboxylic acid or their ester, C-O-H bending mode.	Spherical, hexagonal, nanorod	26 (XRD), agglomeration of 100 (DLS, SEM, TEM)	[89]
Red Rubin basil	*Ocimum basilicum* (Lamiaceae)	Leaf extract	-	Hexagonal (wurtzite)	50 (TEM, EDS), 14.28 (XRD)	[90]
Bhuiamla, stone breaker	*Phyllanthus niruri* (Phyllanthaceae)	Leaf extract	O-H, C-H, C-O stretching, aromatic aldehyde.	Hexagonal wurtzite, quasi-spherical	25.61 (FE-SEM & XRD)	[91]
Buchu	*Agathosma betulina* (Rutaceae)	Dry leaves	O-H of hydroxyl group, Zn-O stretching band	Quasi-spherical agglomerates	15.8 (TEM), 12–26 (HRTEM)	[92]
Red clover	*Trifolium pratense* (Legumes)	Flower	Hydroxyl, -C-O, -C-O-C, C=C stretching mode.	Spherical	60–70 (XRD)	[93]
Kapurli	*Anisochilus carnosus* (Lamiaceae)	Leaf extract	O-H of water, alcohol, phenol C-H of alkane, O-H of carboxylic acid, C=O of the nitro group.	Hexagonal wurtzite, quasi-spherical	56.14 (30 mL of extract), 49.55 (40 mL), 38.59 (50 mL) [XRD], 20–40 (FE-SEM), 30–40 (TEM)	[79]
Water hyacinth	*E. crassipes* (Pontederiaceae)	Leaf extract	-	Spherical without aggregation	32–36 (SEM & TEM), 32 (XRD)	[94]
Dog rose	*Rosa canina* (Rosaceae)	Fruit extract	C-O and C=O of esters, hydroxyl, C-H stretching.	Spherical	[13.3 (CH), 11.3 (MI)] (XRD), [25–204 (CH), 21–243 (MI)] (DLS),	[7]
Black nightshade	*Solanum nigrum* (Solanaceae)	Leaf extract	O-H, aldehydic C-H, amide III bands of protein, carboxyl side group, C-N of amine, the carbonyl group	Wurtzite hexagonal, quasi-spherical	20–30(XRD and FE-SEM),29.79(TEM)	[95]
Aloe vera	*Aloe vera* (Liliaceae)	Freeze-dried leaf peel	-	Spherical, hexagonal	25–65 (SEM & TEM)	[84]
Neem	*Azadirachta indica* (Meliaceae)	Leaf	Amide II was stretching band, C-N stretching band of aliphatic, aromatic amide, an aliphatic amine, alcohol, phenol, secondary amine, C-H of alkane and aromatics, C=C-H of alkynes, C=O, C-C of an alkane.	Spherical	9.6–25.5 (TEM)	[82]
Drumstick tree	*Moringa oleifera* (Moringaceae)	Leaf	O-H, C-H of alkane, C=O of alcohol, carboxylic acid	Spherical and granular nano-sized shape with a group of aggregates	24 (XRD), 16–20 (FE-SEM)	[96]
Coconut	*Cocus nucifera* (Arecaceae)	Coconut water	O-H of alcohol and a carboxylic acid, C=O of ketones, C-N of aromatic and aliphatic amines,	Spherical and predominantly hexagonal without any agglomeration	20–80 (TEM), 21.2 (XRD)	[97]
Cotton	*Gossypium* (Malvaceae)	Cellulosic fiber	O-H, [C=O, C-O, C-O-C] (due to Zn precursor)	Wurtzite, spherical, nanorod	13 (XRD)	[98]
Santa maria feverfew, carrot grass, congress weed	*Parthenium hysterophorus* (Asteraceae)	Leaf extract	N-H bending & N-H stretching mode, a phosphorus compound, secondary sulfonamide, monosubstituted alkyne, amine salt, vinyl cis-tri substituted	Spherical, hexagonal	22–35 (50% plant extract), 75–90 (25% plant extract) (XRD, TEM)	[99]
Neem	*Azadirachta indica* (Meliaceae)	Fresh leaves	O-H between H_2_O and CO_2_, carbonate moieties	Hexagonal disk, nanobuds	10–30 (TEM), 9–40 (XRD)	[83]
Mexican mint	*Plectranthusamboinicus* (Lamiaceae)	Leaf extract	Zn-O, C-O of C-O-SO_3_, phosphorus compound	Rod-shaped nanoparticles with agglomerates	50–180 (SEM)	[100]
Crown flower	*Calatropis gigantea* (Apocynaceae)	Fresh leaves	-	Spherical-shaped forming agglomerates	30–35 (SEM)	[101]
Nochi	*Vitex negundo* (Lamiaceae)	Flowers	-	Hexagonal	38.17 (XRD), 10–130 (DLS)	[30]
Sandalwood	*S. album* (Santalaceae)	Leaves	N-H stretching of amide II, carboxylate group, carbonyl stretching, O-H of alcohol	Nano rods	100 (DLS & SEM), 70–140 (TEM)	[102]
Nochi	*Vitex negundo* (Lamiaceae)	Leaf	OH, C-H, C=C stretching band.	Spherical	75–80 (SEM & EDX), 38.17 (XRD)	[103]
Rambutan	*Nephelium lappaceum* (Sapindaceae)	Fruit peels	O-H stretching, H-O-H bending	Needle-shaped forming agglomerate	50.95 (XRD)	[104]
Aloe Vera	*Aloe Vera* (Liliaceae)	Leaf extract	O-H of phenol, amines, O-H of alcohol, and C-H of alkanes, the amide of protein and enzymes.	Spherical, oval, hexagonal	8–20 (XRD)	[85]
African tulip tree	*Sphathodea campanulata* (Bignoniaceae)	Leaf extract	O-H stretching of polyphenols, nitrile group, C-H, C=O group	Spherical	30–50 (TEM)	[105]

**Table 2:** Synthesis of ZnO-NP using bacterial strain.

Family	Bacterial Strain	Functional Group	Shape	Size (nm)	References
Bacillaceae	*Lactobacillus sporogens*	-	Hexagonal unit cell	5–15 (TEM), 11 (XRD)	[112]
Pseudomonadaceae	*Pseudomonas aeruginosa*	O-H stretching vibration, -CH of aliphatic stretching vibration, ester carbonyl group.	Spherical	35–80 (TEM), 27 (XRD), 81 (DLS)	[113]
Pseudomonadaceae	*Aeromonas hydrophila*	Phosphorus compound, vinyl cis-trisubstituted, monosubstituted alkyne	Spherical, oval	57.72 (AFM), 42–64 (XRD)	[114]
Bacillaceae	*B.licheniformis*	0-H, N-H,-C-O (carbonyl stretching in the amide I and amide II linkage of protein), C-N stretching bond.	Nanoflowers	200 with nanopetals 40 in width and 400 in length (TEM)	[108]
Nocardiaceae	*Rhodococcus pyridinivorans*	Phosphorus compound, secondary sulfornamide, monosubstituted alkyne, β-lactone, amine salt, amide II stretching band, enol of 1-3-di ketone, a hydroxy aryl ketone, amide I bending band, alkane, mononuclear benzene band.	Hexagonal phase, roughly spherical	100–120 (FE-SEM), 120–130 (XRD)	[110]
Enterobacteriacea	*Serratia ureilytica* (HM475278)	-	Spherical- to nanoflower-shaped	170–250 (30 min), 300–600 (60 min), 185–365 (90 min) [SEM]	[115]

**Table 3:** Synthesis of ZnO-NPs using algae.

Algal Strain	Family	Size (nm)	Shape	Functional Group	Reference
*Chlamydomonas reinhardtii*	Chlamydomonaceae	55–80 (HR-SEM), 21 (XRD)	Nanorod, nanoflower, porous nanosheet	C=O stretching, N-H bending band of amide I and amide II, C=O stretch of zinc acetate, C-O-C of polysaccharide	[117]
*S. myriocystum*	Sargassaceae	46.6 (DLS), 20–36 (AFM)	Spherical, radial, triangle, hexagonal, rod	O-H and C=O stretching band, carboxylic acid	[118]
*Sargassum muticum*	Sargassaceae	30–57 (FE-SEM), 42 (XRD)	Hexagonal wurtzite	Asymmetric stretching band of the sulfate group, an asymmetric C-O band associated with C-O-SO_3_ & -OH group, sulfated polysaccharides	[119]

**Table 4:** Synthesis of ZnO NPs using fungi.

Family	Fungal Strain	Functional Group	Shape	Size (nm)	Reference
Trichocomaceae	*Aspergillus* strain	-	Spherical forming aggregates	50–120 (SEM)	[125]
Trichocomaceae	*Aspergillus terreus*	C-N bond of primary amine, C-O of a primary alcohol, primary and secondary alcohol, N=O aromatic nitro compound, alkyl C=C, amide, open-chain amino group	Spherical	54.8–82.6 (SEM), 29 (XRD)	[126]
	*Candida albicans*	-	Quasi-spherical, hexagonal phase (wurtzite structure)	25 (XRD), 15–25 (SEM), 20 (TEM)	[124]
Trichocomaceae	*Aspergillus fumigatus* TFR-8	-	Oblate spherical and hexagonal forming aggregates	1.2–6.8 (DLS), 100 (agglomerate)	[106]

**Table 5:** Synthesis of ZnO-NPs by proteins.

Others	Size (nm)	Shape	Functional Group	References
Egg albumin	16 (XRD), 10–20 (TEM), 8–22 (AFM)	Spherical, Hexagonal wurtzite	Hydroxyl group	[129]
L-alanine	50–110 (TEM, SEM)	-	Hydroxyl group, C-O vibration of Schiff- base.	[130]
Soluble starch	50 (SEM)	-	-	[131]

**Table 6:** Green-synthesized ZnO-NPs applications for antibacterial purposes.

Platform	Raw Material	Size	System	Targeted Bacteria	Reference
Bacteria-mediated	*Bacillus megaterium*	45–95 nm	ZnO-NPs	*H. pylori*	[144]
	*Bacillus licheniformis*	10–100 nm	ZnO-NPs	*P. aeruginosa* *Proteus vulgaris* *Bacillus subtilis* *Bacillus pumilus*	[145]
Plant-mediated	*Cassia fistula*	5–15 nm	ZnO-NPs	*Klebsiella aerogenes* *E. coli* *Plasmodium desmolyticum*	[146]
	*Trifolium pretense*	60–70 nm	ZnO-NPs	*P. aeruginosa* *E. coli* *S. aureus*	[93]
	*Boerhavia diffusa*	140 nm	ZnO-NPs	*MRSA*	[147]
	*Artocarpus gomezianus*	39, 35, 31 nm prepared with 5, 10 and 15 mL of 10% extract	ZnO-NPs	*S. aureus*	[148]
	*Sechium edule*	30–70 nm	ZnO-NPs	*Bacillus subtilis* *Klebsiella pneumonia*	[149]
	*Azadirachta indica*	9.6–25.5 nm	ZnO-NPs	*Streptococcus pyogenes* *E. coli* *S. aureus*	[82]
	*Azadirachta indica*	9–40 nm	ZnO-NPs	*Klebsiella aerogenes* *S. aureus*	[83]
	*Acalypha indica*	20 nm	ZnO-NPs	*E. coli* *S. aureus*	[150]
	*Tabernaemontana divaricata*	20–50 nm	ZnO-NPs	*E. coli* *S. aureus* *Salmonella paratyphi*	[151]
	*Laurus nobilis*	47.27 nm	ZnO-NPs	*P. aeruginosa* *S. aureus*	[152]
	*Ruta graveolens*	28 nm	ZnO-NPs	*Klebsiella aerogenes* *P. aeruginosa* *E. coli* *S. aureus*	[31]
	*Aristolochia indica*	22.5 nm	ZnONPs	Multi-drug resistant organisms (MDROs) isolated from pus samples of DFU patients	[153]
	*Allium sativum*	14 and 27 nm	ZnO-NPs	*S. aureus* *Bacillus subtilis* *L. monocytogenes* *E. coli* *Salmonella typhimurium* *P. aeruginosa*	[153]
	*Bauhinia tomentosa*	22–94 nm	ZnO-NPs	*E. coli* *P. aeruginosa*	[154]
	*Ulva lactuca*	10–50 nm	ZnO-NPs	*Bacillus licheniformis* *Bacillus pumilis* *E. coli* *Proteus vulgaris*	[155]
	*Amaranthus spinosus*	243 nm undoped/197 nm 1%-Fe-ZnO-NPs	Undoped and Fe-doped ZnO-NPs	*E. coli* *Bacillus safensis*	[156]
	*Hibiscus rosa-sinensis*	15–170 nm	Fe-doped ZnO-NPs	*E. coli*	[157]
	*G. sylvestre*	138 nm, 52 nm, 59 nm, and 63 nm for undoped, La-, Ce-, and Nd-doped	Lanthanum-, cerium-, and neodymium-doped ZnO-NPs	*S. aureus* *Streptococcus pneumonia*	[158]

**Table 8:** ZnO-NPs-based drug delivery methods.

Materials	Cell Line	Drug	References
ZnO (Tetrapod)	CHO-K1, HeLa, Vero, VK2/E6	-	[204]
ZnO@PMAA-co-PDMAEMA-NPs	COS-7	DNA	[205]
ZnO/Carboxymethyl Cellulose (CMC)	L929, MA104	Curcumin	[206]
Curcumin/O-CMCS/n-ZnO nanocomposites	MA 104	Curcumin	[207]
Mesoporous ZnO	-	DOX	[208]
ZnO@PNIPAM-NPs	-	DOX	[209]
ZnO-NPs	T47D	PPDME	[210]
ZnO-NPs	HeLa	DOX	[211]
ZnO/PEG-NPs	Gram-positive microorganisms	DOX	[212]
ZnO/Au-NPs	Hela	Camptothecin	[213]
ZnO-QDs	HepG2	-	[214]
Chitosan/ZnO-NPs	-	DOX	[201]
ZnO	cancerous T, activated human T	-	[215]
ZnO@Polymer-NPs	U251	DOX	[216]

**Table 9:** Bioimaging Uses of ZnO-NPs.

Model	Type of Material	Size (nm)	Reference
Skin tissue/cellular architecture	ZnO-NPs	15–30	[218]
KB cells	ZnO Nanocrystals	<100	[219]
*S. oneidensis*	CdSe(S)/ZnO-QDs	2–4	[220]
Human skin and rat liver cells	ZnO-NPs	26–30	[221]
Plants tissues cell implosion	ZnO-NPs	2–200	[222]
Blood cells of zebrafish; roots and shoots of *Arabidopsis* plants	ZnO-NPs	10–300	[223]
Hela cells	ZnO@silica-NPs	2–5	[224]
Skin	ZnO-NPs	21	[225]
B16F10 cells	ZnO/Au@PEG-NPs	45–98	[226]

## Data Availability

Available data are presented in the manuscript.
